# Artificial intelligence-based magnetic resonance imaging for preoperative staging of patients with endometrial cancer: a systematic review and meta-analysis

**DOI:** 10.3389/fonc.2025.1673060

**Published:** 2026-01-05

**Authors:** Jinjing Zheng, Xueyao Lin, Ming Li

**Affiliations:** Department of Radiology, Ningbo Medical Center Lihuili Hospital, Ningbo University, Ningbo, Zhejiang, China

**Keywords:** artificial intelligence, magnetic resonance imaging, endometrial cancer, meta-analysis, systematic review

## Abstract

**Objective:**

To systematically collect all literature on the value of artificial intelligence (AI)-based magnetic resonance imaging (MRI) for preoperative prediction of myometrial invasion and cervical stroma invasion in patients with endometrial cancer and conduct a meta-analysis to provide the latest and most comprehensive synthesis of current research findings.

**Methods:**

A systematic literature search was conducted in PubMed, Web of Science, Embase, and the Cochrane Library databases up to March 2025. The methodological quality of the included studies was assessed using the QUADAS-2 tool. Statistical analyses were primarily conducted using Stata15.0 and Review Manager 5.4.1 software. Outcomes included combined Sen, Spe, +LR, -LR, DOR, and their 95% CI. The SROC curve was plotted, and the AUC was calculated. The Deeks’ funnel plot was used to detect publication bias and assumed small-study effects.

**Results:**

Finally, 8 studies (including 13 cohorts) were included. The overall performance of AI-based MRI for the prediction of deep myometrial invasion showed a combined Sen, Spe, +LR, -LR, DOR, and AUC value of 0.80 (95% CI: 0.75-0.85), 0.81 (95% CI: 0.64-0.91), 4.2 (95% CI: 2.0-8.5), 0.24 (95% CI: 0.17-0.34), 17 (95% CI: 6-47), and 0.83 (95% CI: 0.80-0.86), respectively. The overall performance of AI-based MRI for the prediction of cervical stroma invasion showed a combined Sen, Spe, +LR, -LR, DOR, and AUC value of 0.78 (95% CI: 0.55-0.91), 0.86 (95% CI: 0.79-0.91), 5.6 (95% CI: 4.3-7.4), 0.25 (95% CI: 0.12-0.55), 22 (95% CI: 11-44), and 0.90 (95% CI: 0.87-0.92) respectively.

**Conclusion:**

AI-based MRI can improve the accuracy of preoperative staging of patients with endometrial cancer to a certain extent. However, considering the limitations of this article, additional large-scale, prospective, multicenter clinical trials are necessary to further investigate the utility of AI-based MRI in the preoperative staging of endometrial cancer.

## 1 Introduction

Endometrial cancer comprises a group of epithelial malignancies originating from the endometrium. Endometrioid carcinoma is the most common histologic type and is ranked among the three major malignant tumors of the female reproductive system. Its incidence continues to rise each year. In the United States and other countries, the incidence has exceeded that of cervical cancer and ovarian cancer (1–3). The global incidence rate of endometrial cancer was approximately 8.7 per 100,000 women in 2022, with nearly 420,000 new cases diagnosed annually worldwide (4). It is well known that the treatment of endometrial cancer includes surgical resection and adjuvant chemoradiotherapy. The choice of treatment and patient prognosis are influenced by multiple factors such as age, histological type, pathological grade, degree of cervical invasion, and lymph node metastasis (5). For younger patients, accurate staging is essential to guide clinicians in developing surgical plans to preserve fertility. Different types of cancer have different sensitivity to treatment and prognosis. Cancers with high pathological grades usually show invasive growth and poor prognosis, so more aggressive treatment strategies may be required. If cancer cells invade the cervical area or lymph nodes, treatment plans may differ and involve more extensive surgery or chemoradiotherapy. Therefore, it is particularly important to accurately diagnose the patient before treatment (6, 7).

Magnetic resonance imaging (MRI) can be used for both conventional sequence imaging and functional imaging. It has a high resolution for soft tissue and can distinguish between the uterine mucosa, submucosa, myometrium, and serosa (8, 9). MRI has a high clinical value in preoperatively evaluating the depth of myometrial invasion and pelvic iliac and abdominal lumbar lymph nodes metastasis in individuals diagnosed with endometrial cancer. To a certain extent, preoperative staging determines the choice of surgical scope for patients. When judging the degree of cancer invasion, the depth of myometrial invasion is a very important reference indicator because it is related to whether there is metastasis to the pelvic iliac and abdominal lumbar lymph nodes and will affect the patient’s 5-year survival rate (10). According to literature reports, when cancer cells do not infiltrate the myometrium or infiltrate less than 50% of the myometrium, the proportion of lymph nodes invaded by tumor cells is low, and the 5-year survival rate of such patients is high; when endometrial cancer infiltrates 50% or more of the myometrium, the lymph node metastasis rate of these patients will increase (11), and the 5-year survival rate will be significantly lower than that of patients who do not infiltrate the myometrium or only infiltrate less than 50% of the myometrium (12). From this, we can see that MRI can provide corresponding guidance for clinical staging treatment, but MRI examinations have certain error rates and misdiagnosis rates in terms of sensitivity and accuracy and need to be combined with other auxiliary examinations for joint evaluation.

Over the past few years, with the continuous development and application of artificial intelligence (AI), MRI-based deep learning models have shown great application prospects in the field of medical imaging diagnosis. AI reconstructs and analyzes image data in a structured manner, extracts key features of lesions, establishes computer vision training and recognition models, and then generates disease analysis reports and auxiliary diagnosis plans (13–15). AI imaging diagnosis has achieved great results in endoscopic diagnosis of gastric cancer (16), MRI diagnosis of colorectal cancer (17), CT diagnosis of small lung nodules (18), and mammography diagnosis of breast cancer (19). Related studies have used AI technology to read fine features in images, classify images and predict diagnosis (20). In addition, preoperatively, the presence of lymphovascular space invasion, lymph node metastasis, etc., can be predicted based on the images, thereby guiding individualized treatment (21, 22). To date, many AI-based MRI technologies have been used for preoperative staging of endometrial cancer, but no relevant meta-analysis has been reported, and their exact sensitivity and specificity have not yet been determined. Therefore, this study systematically retrieved all relevant literature and performed a meta-analysis to compile the most current and extensive scientific data to assess the effectiveness of AI-based MRI for preoperative staging of endometrial cancer.

## 2 Methods

### 2.1 Protocol and registration

This meta-analysis and systematic review was conducted in accordance with the PRISMA 2020 guidelines (23) and was prospectively registered in the PROSPERO database (CRD420251059395).

### 2.2 Search strategy

We conducted a systematic search of literature published in PubMed, Web of Science, Embase, and Cochrane Library databases from their inception to March 2025, employing both MeSH terms and free-text keywords such as: “Artificial Intelligence”, “Magnetic Resonance Imaging”, and “Endometrial Neoplasms”. The search strategies for PubMed were as follows: (((“Artificial Intelligence”[Mesh]) OR ((((((((Computer Reasoning) OR (Machine Intelligence)) OR (Computational Intelligence)) OR (Computer Vision Systems)) OR (Computer Vision System)) OR (Knowledge Acquisition)) OR (Knowledge Representation)) OR (Knowledge Representations))) AND ((“Magnetic Resonance Imaging”[Mesh]) OR ((((MRI) OR (NMR Imaging)) OR (MR Tomography)) OR (NMR Tomography)))) AND ((“Endometrial Neoplasms”[Mesh]) OR (((((Endometrial Neoplasm) OR (Endometrial Carcinoma)) OR (Endometrium Cancer)) OR (Endometrium Carcinoma)) OR (Endometrial Cancer))). In addition, the reference lists of all selected studies were examined manually to identify any potentially relevant articles. Two independent reviewers carried out the study selection process. Discrepancies in study inclusion were resolved through discussion and consensus. All identified articles were organized and verified using EndNote X9.

### 2.3 Inclusion and exclusion criteria

Both prospective and retrospective studies investigating the role of AI-enhanced MRI in preoperative staging of endometrial cancer were considered in this analysis.

Inclusion criteria:

Study population: patients diagnosed with endometrial cancer.
Diagnostic criteria: the experiment to be evaluated is AI-based MRI, with no restrictions on the model or methodology. The gold standard is histopathology.
Outcomes: sensitivity (Sen), specificity (Spe), positive likelihood ratio (+LR), negative likelihood ratio (-LR), diagnostic odds ratio (DOR), pre-test probability, post-test probability, summary receiver operating characteristic (SROC) area under the curve (AUC), etc.
Study design: prospective and retrospective studies that focused on the value of AI-based MRI for preoperative staging of patients with endometrial cancer.

Exclusion criteria: (1) duplicate publications; (2) articles unrelated to this study; (3) reviews, meta-analyses, conferences, letters, comments, etc.; (4) studies involving animal models; (4) articles lacking sufficient or incomplete data; (5) non-English language publications. Two researchers (Zheng JJ and Lin XY) independently screened the literature. Any disagreements during the screening process were first resolved through discussion and negotiation between the two researchers. If no agreement could be reached after discussion, a third researcher (Li M) was invited to arbitrate, and the arbitrator’s opinion became the final decision.

### 2.4 Data extraction

Two reviewers independently collected relevant data from each eligible study, including details such as the first author’s name, publication year, study design, participants’ age and gender, AI models, reference standard, sample size, true positives (TP), false positives (FP), true negatives (TN), false negatives (FN), Sen, Spe, +LR, -LR, DOR, and AUC value, etc. Disagreements in data collection were resolved through consultation with a third reviewer. If specific information was lacking, attempts were made to contact the corresponding authors for clarification or additional data. All collected variables were organized using Microsoft Excel. Any disagreements during the data extraction process will first be resolved through discussion and negotiation between the two parties. If no agreement can be reached after discussion, a third researcher (Li M) will be invited to arbitrate, and the arbitrator’s opinion will be the final decision.

### 2.5 Quality assessment

In accordance with the methodological recommendations provided in the Cochrane Handbook for systematic reviews of diagnostic trials, we assessed the methodological quality of the included studies using the QUADAS-2 tool. Subsequently, the Review Manager 5.4.1 software was employed to present the final evaluation findings in a comprehensive manner (24). The quality of the included studies was independently evaluated by two reviewers, and any discrepancies were resolved through discussion or, if needed, consultation with a third reviewer. The QUADAS-2 evaluates study quality by examining two aspects: risk of bias and applicability to clinical practice. Risk of bias is assessed across four domains: patient selection, index test, reference standard, and flow and timing. Applicability concerns are judged based on three domains: patient selection, index test, and reference standard. Each domain related to risk of bias includes multiple signaling questions, with answer options of “yes/no/unclear”. If the answers to all signaling questions are “yes”, then that aspect is regarded as having a low probability of bias. If any response is marked as “no”, it may indicate a potential risk of bias. There are no signaling questions for clinical applicability, only an overall assessment, with answer options including “high risk/low risk/unclear”. It is worth noting that the “unclear” response should be chosen when the information provided in the literature is incomplete during the assessment process.

### 2.6 Data analysis

Data analysis was conducted using Stata version 15.0 and Review Manager 5.4.1. A bivariate random-effects model was employed to calculate pooled Sen, Spe, +LR, −LR, and DOR with 95% CIs. SROC curves were constructed, and AUC values were determined accordingly. The χ² test was applied to assess statistical heterogeneity across the included studies, and the significance level was set at α = 0.05. Heterogeneity was further quantified using the I² statistic. If I² ≤ 50%, heterogeneity was considered low; if I² > 50%, it was regarded as high. Deeks’ funnel plot was applied to evaluate publication bias and detect potential small-study effects, with a P value below 0.05 indicating the presence of bias in the analyzed studies.

## 3 Results

### 3.1 Literature screening process and results

**Figure 1:**
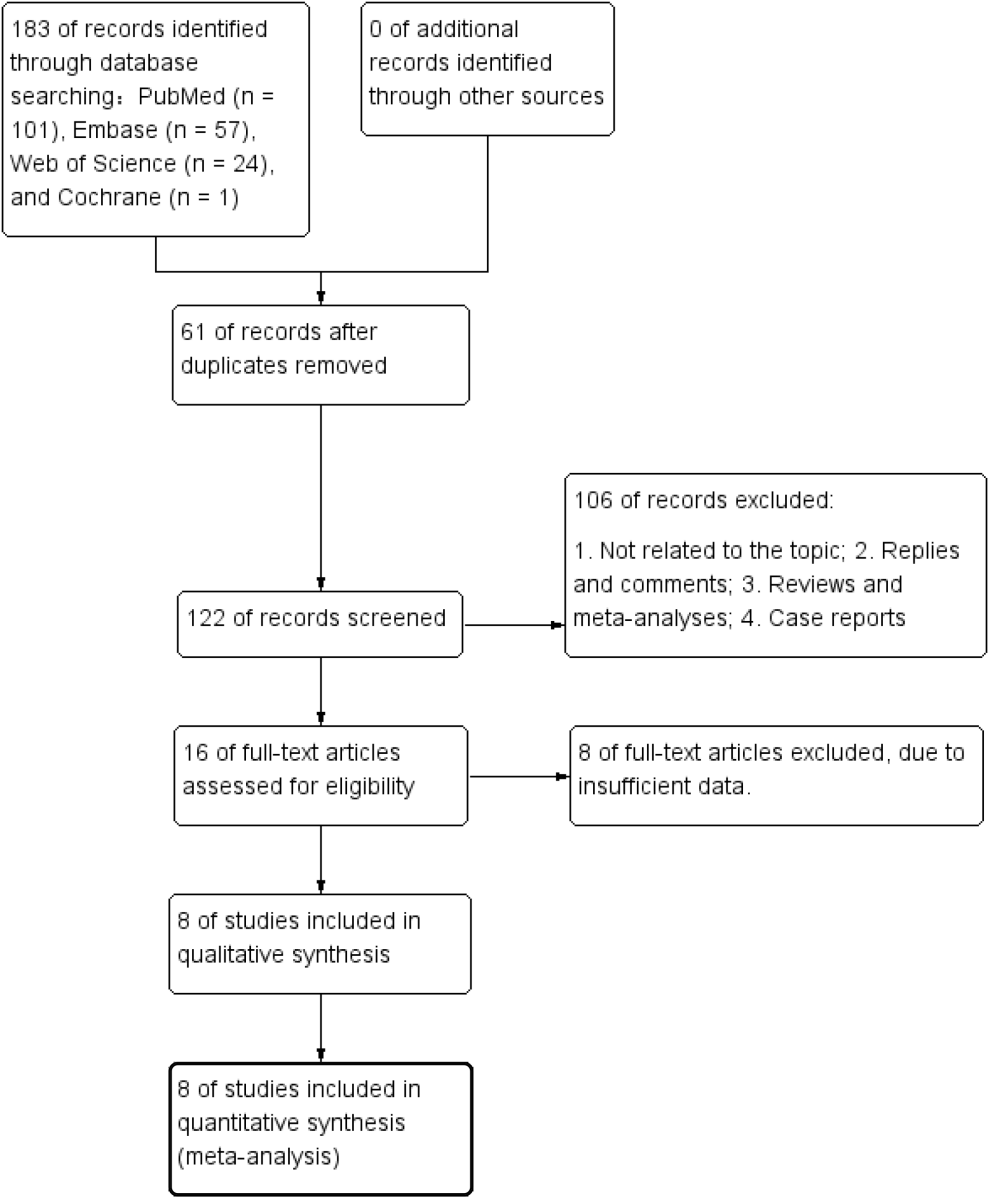
PRISMA flow-chart for the systematic review and meta-analysis.

According to the literature search strategy, 183 relevant publications were retrieved. Following the removal of duplicates using EndNote X9, 122 articles remained. After screening titles and abstracts, 106 publications were excluded, resulting in 16 articles for initial screening. Of the 16 articles assessed in full, 8 were excluded due to insufficient information or failure to meet inclusion criteria. The study ultimately included a total of 8 articles (25–32). **Figure 1** displays the screening procedure for the selected literature, and **Table 1** summarizes the key characteristics of the studies included in the review. If there were multiple models or multiple cohorts in the same study, they were extracted separately and marked by adding the letters “a, b, c, or d, etc.” after the name of studies.

**Table 1:** Characteristics of include studies.

Study	Country	Study design	AI model	Golden standard	Sample size	Mean/median age
Chen 2020 (25)	China	Retrospective	YOLOv3 algorithm	Pathological diagnosis	138	53.5
Lecointre 2025 (26)	France	Retrospective	Deep Learning Model	Pathological diagnosis	178	75
Lefebvre 2023 (27)	Canada and France	Retrospective	Spherical harmonics	Pathological diagnosis	53	67
Lefebvre 2023 (27)	Canada and France	Retrospective	Radiomics	Pathological diagnosis	53	67
Lefebvre 2022 (28)	Canada and France	Retrospective	Pyradiomics 3.0	Pathological diagnosis	63	67
Rodríguez-Ortega 2021 (29)	Spain	Retrospective	Four Adaboost models	Pathological diagnosis	143	64.7
Tao 2022 (30)	China	Prospective	ResNet network	Pathological diagnosis	80	NA
Wang 2025a (21)	China	Retrospective	Radiomics	Pathological diagnosis	182	52.7
Wang 2025b (31)	China	Retrospective	Deep learning	Pathological diagnosis	182	52.7
Wang 2025c	China	Retrospective	Stacking	Pathological diagnosis	182	52.7
Wang 2025d	China	Retrospective	Ensemble	Pathological diagnosis	182	52.7
Wang 2024a (32)	China	Retrospective	Internal test	Pathological diagnosis	567	54.03
Wang 2024b	China	Retrospective	External test	Pathological diagnosis	567	54.03

### 3.2 Quality assessment of included studies

**Figure 2:**
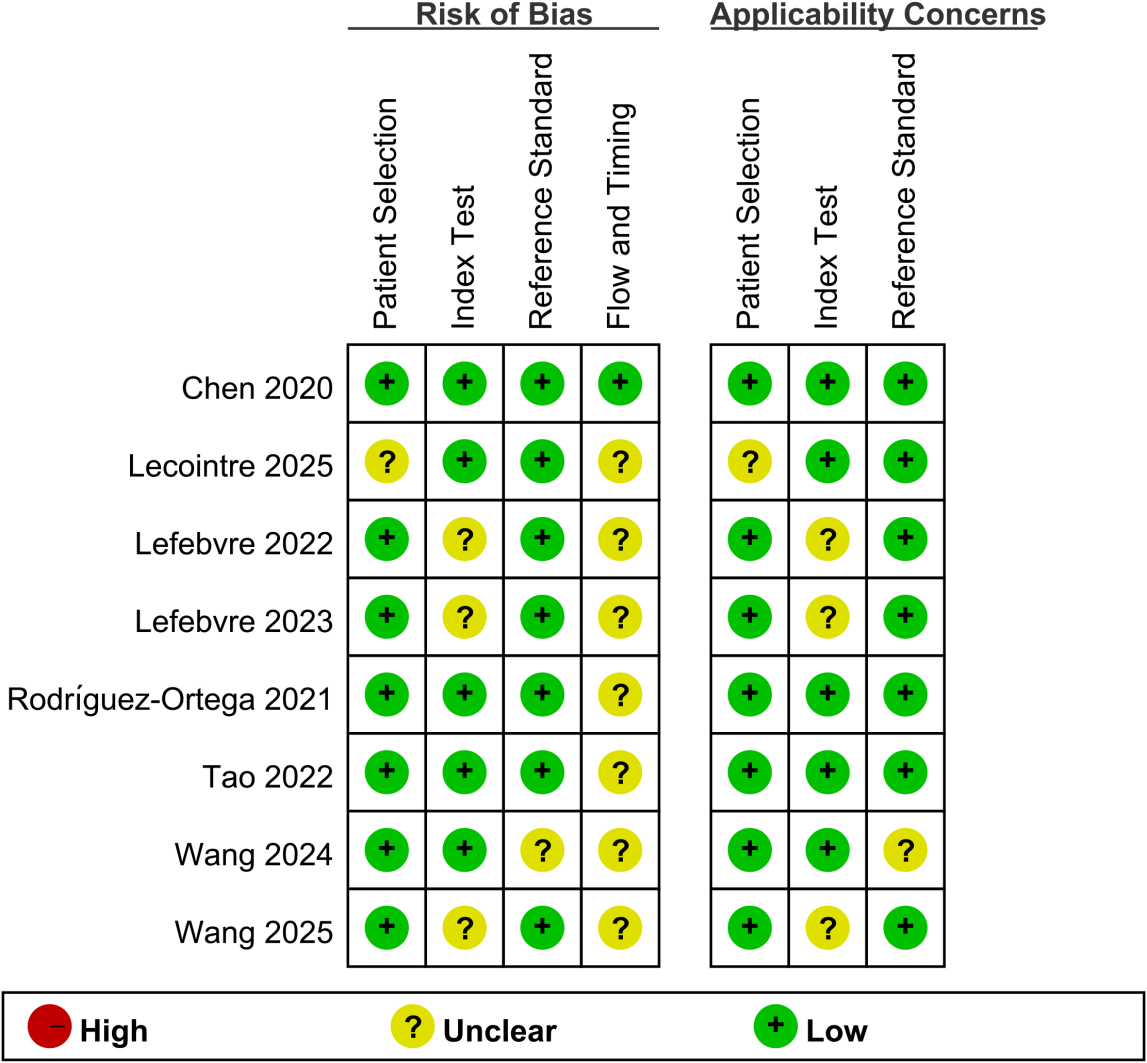
Risk of bias and applicability concerns summary.

For the assessment of risk of bias, the QUADAS-2 tool was employed. **Figure 2** presents the results of the methodological quality evaluation of the analyzed studies. In general, the publications analyzed were of high quality, with most exhibiting either a low or unclear risk of bias.

### 3.3 Meta-analysis of deep myometrial invasion

**Figure 3:**
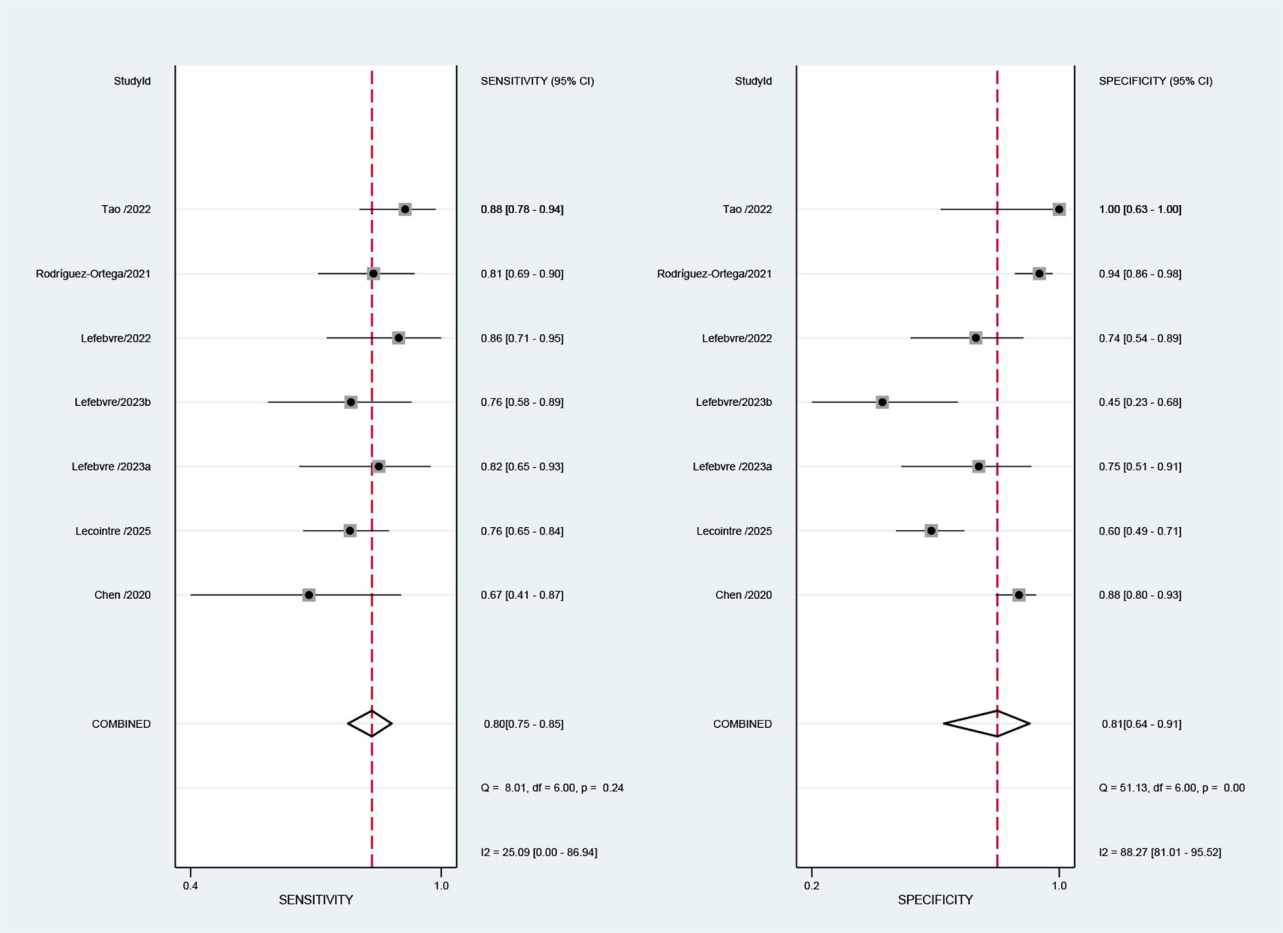
Forest plots illustrate the pooled sensitivity and specificity of AI-based MRI prediction of deep myometrial invasion in endometrial cancer. A random-effects model was used for pooling. Estimates from each study are represented by squares, with size representing weight, and horizontal lines representing 95% confidence intervals. Pooled statistics are represented by diamonds. The I² statistic was used to quantify heterogeneity.

**Figure 4:**
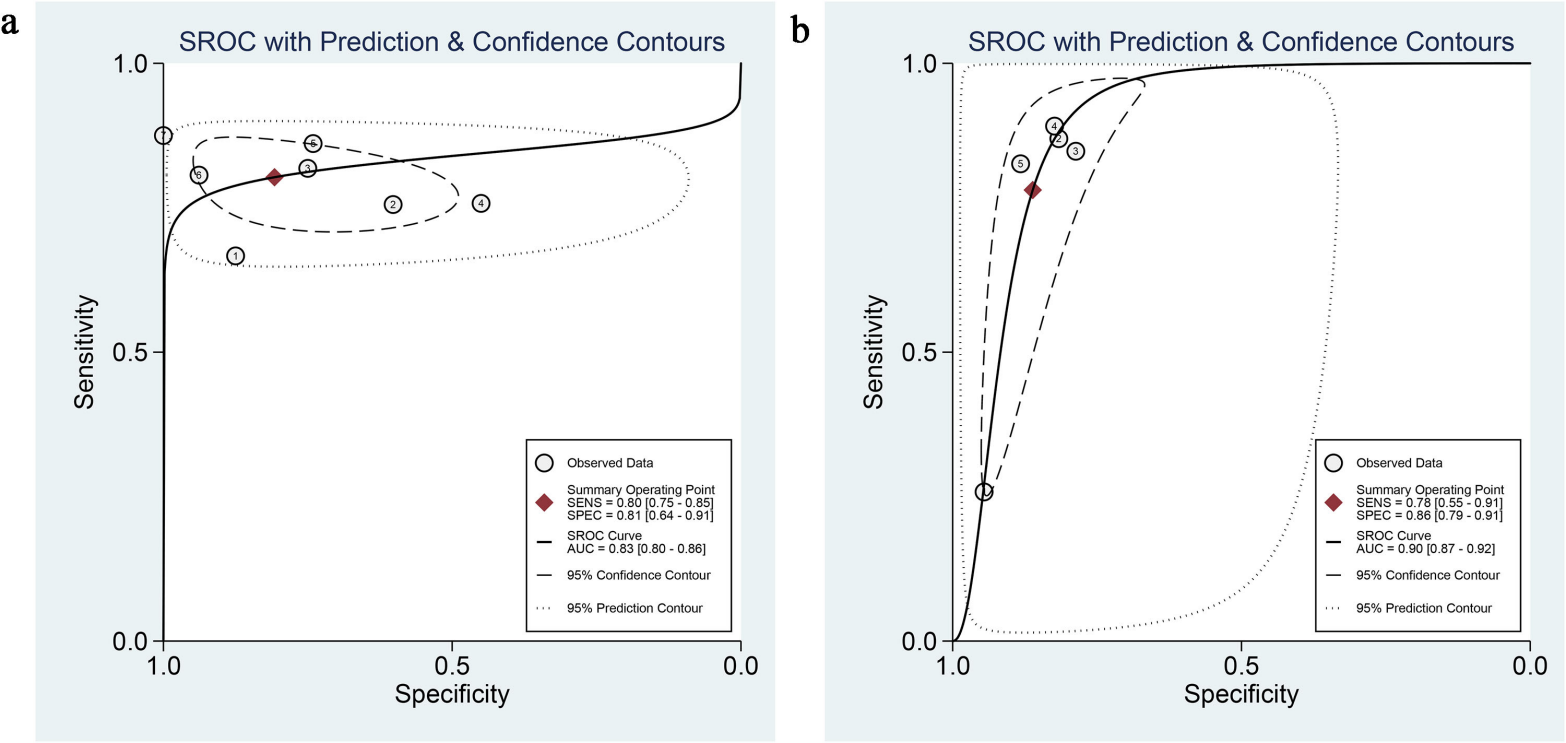
AI-based SROC curves for MRI prediction of **(A)** deep myometrial invasion and **(B)** cervical stromal invasion. The figure shows the pooled sensitivity and specificity (solid circles), SROC curves (solid lines), 95% confidence region (dashed lines), and 95% prediction region (dotted lines). AUC values ​​and their 95% CIs are given.

A total of six studies (including seven cohorts) reported the value of AI-based MRI for the detection of deep myometrial invasion for endometrial cancer. The overall performance of AI-based MRI for the prediction of deep myometrial invasion showed a combined Sen, Spe, +LR, -LR, and DOR of 0.80 (95% CI: 0.75-0.85), 0.81 (95% CI: 0.64-0.91), 4.2 (95% CI: 2.0-8.5), 0.24 (95% CI: 0.17-0.34), and 17 (95% CI: 6-47), respectively (**Figure 3**). Considerable heterogeneity in specificity was observed in the aggregated forest plot results (*I*^2^ = 88.27%; 95% CI: 81.01-95.52%) (**Figure 3**), whereas the heterogeneity in sensitivity was relatively lower (I²=25.09%; 95% CI: 0.00-86.94%) (**Figure 3**). **Figure 4A** represented the SROC with prediction and confidence contours, with an AUC of 0.83 (95% CI: 0.80-0.86).

### 3.4 Meta-analysis of cervical stroma invasion

**Figure 5:**
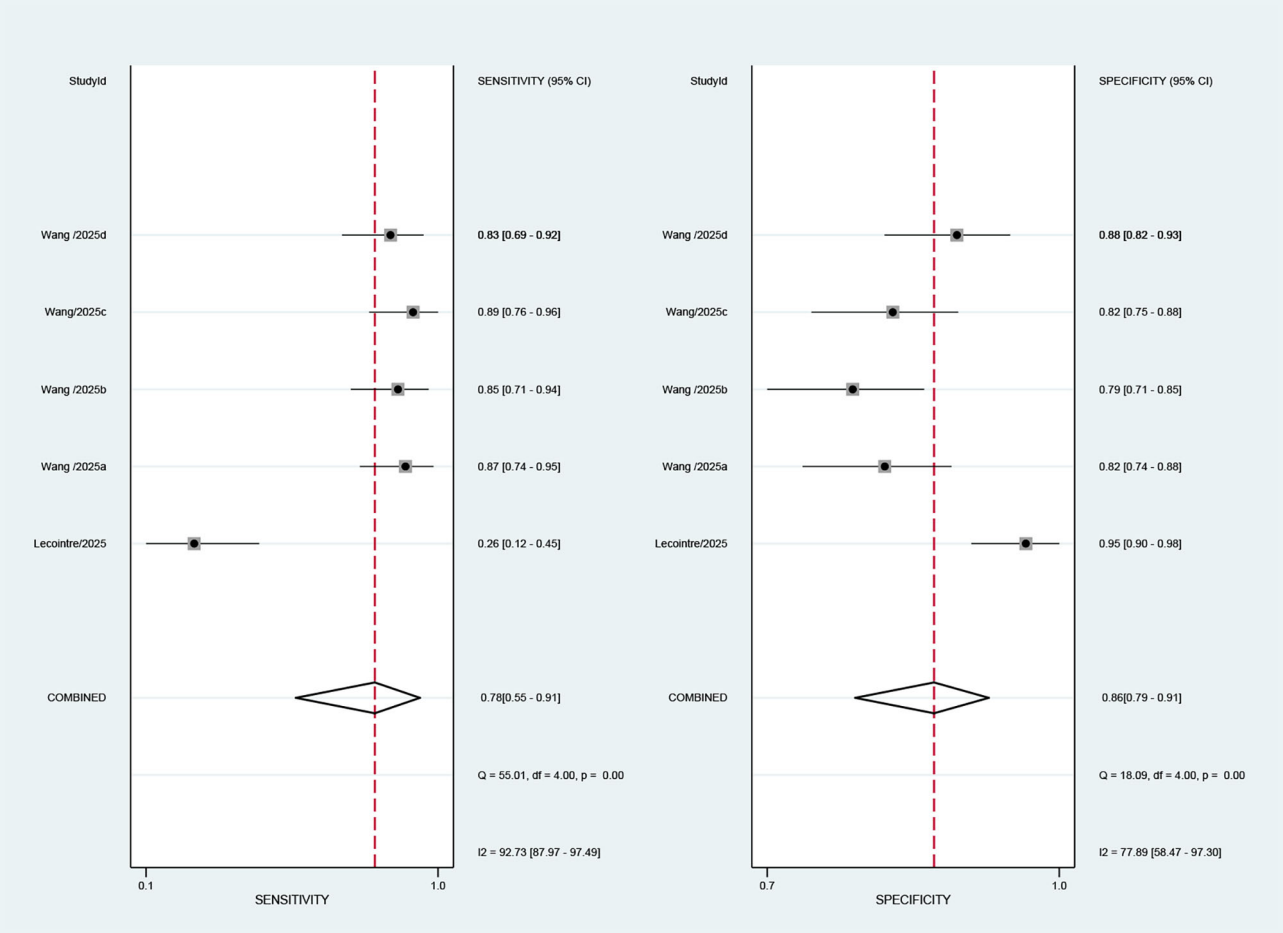
Forest plots illustrate the pooled sensitivity and specificity of AI-based MRI prediction of cervical stromal invasion in endometrial cancer. A random-effects model was used for pooling. Estimates from each study are represented by squares, with size representing weight, and horizontal lines representing 95% confidence intervals. Pooled statistics are represented by diamonds. The I² statistic was used to quantify heterogeneity.

A total of two studies (including five cohorts) reported the value of AI-based MRI for the detection of cervical stroma invasion for endometrial cancer. The overall performance of AI-based MRI for the prediction of cervical stroma invasion showed a combined Sen, Spe, +LR, -LR, and DOR of 0.78 (95% CI: 0.55-0.91), 0.86 (95% CI: 0.79-0.91), 5.6 (95% CI: 4.3-7.4), 0.25 (95% CI: 0.12-0.55), and 22 (95% CI: 11-44), respectively (**Figure 5**). It is important to note that the 95% confidence interval of its sensitivity is relatively wide, suggesting that this estimate is subject to significant uncertainty and should be interpreted with extreme caution. Substantial heterogeneity in sensitivity was observed across the forest plot analysis (*I*^2^ = 92.73%; 95% CI: 87.97-97.49%) and specificity (*I*^2^ = 77.89%; 95% CI: 58.47-97.30%) (**Figure 5**). **Figure 4B** illustrates the SROC with prediction and confidence contours, with an AUC of 0.90 (95% CI: 0.87-0.92).

### 3.5 Publication bias

**Figure 6:**
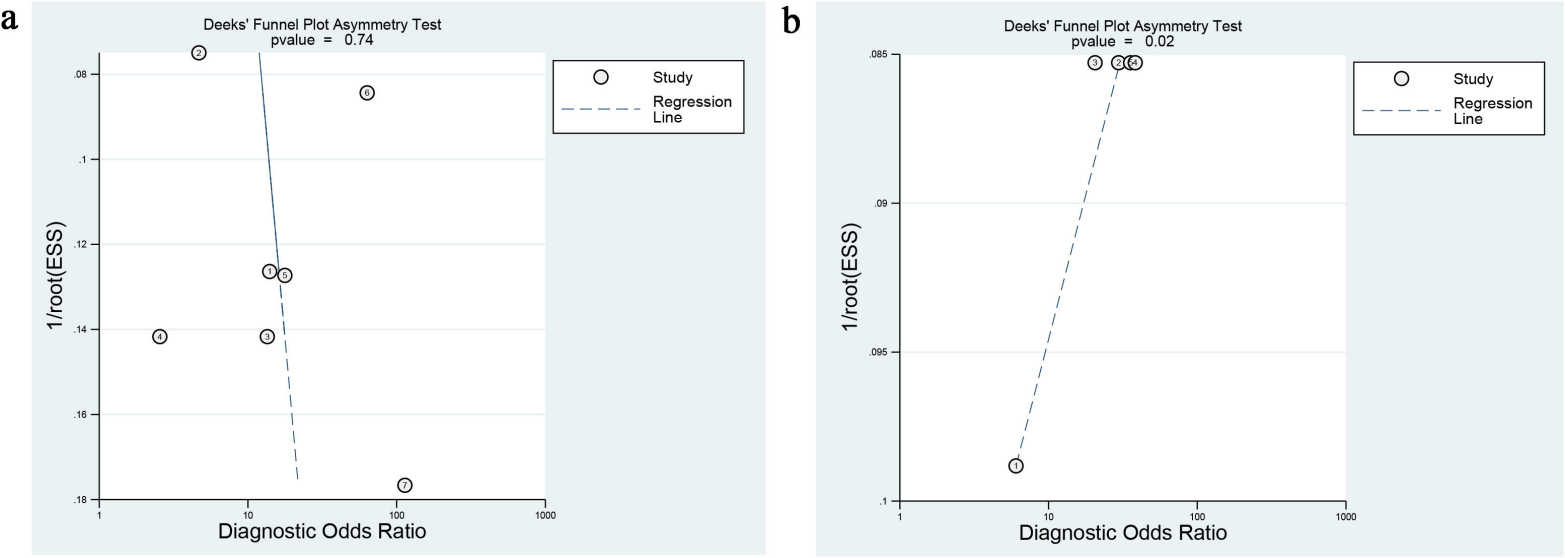
Deeks’ funnel plots are used to assess publication bias in analyses of **(A)** deep myometrial invasion and **(B)** cervical stromal invasion. In the absence of significant publication bias, study points should be approximately symmetrically distributed around the regression line. The p-value is used to test the significance of asymmetry.

Deeks’ test was employed to assess publication bias in the included studies, yielding a *P* value of 0.74 for deep myometrial invasion (**Figure 6A**) and 0.02 of cervical stromal invasion (**Figure 6B**), suggesting that the meta-analysis of cervical stroma invasion was affected by significant publication bias.

## 4 Discussion

In current clinical practice, preoperative assessment of local invasion of endometrial cancer typically involves a combination of transvaginal ultrasound and MRI (33). MRI is valuable in assessing the depth of myometrial invasion and cervical stromal invasion due to its superior soft tissue resolution. Furthermore, ultrasound also demonstrates high accuracy in detecting cervical invasion, and its accuracy is comparable to MRI when performed by an experienced operator (34). However, the diagnostic accuracy of both ultrasound and MRI depends largely on the experience of the operator or interpreter, and visual assessment can be subjective. Therefore, exploring artificial intelligence technology as an objective and reliable auxiliary tool to improve the accuracy and consistency of preoperative imaging assessment is of significant clinical significance.

This research conducted a comprehensive systematic review and meta-analysis of clinical data on the preoperative staging of endometrial cancer patients using AI-based MRI technology to obtain the combined diagnostic efficacy and comprehensively assess the diagnostic value of AI-based MRI for endometrial cancer patients. The results of this study indicated that the combined Sen, Spe, +LR, -LR and DOR of deep myometrial invasion detected by AI-based MRI were 0.80, 0.81, 4.2, 0.24 and 17, respectively. Additionally, the combined Sen, Spe, +LR, -LR and DOR of cervical stroma invasion detected by AI-based MRI were 0.78, 0.86, 5.6, 0.25 and 22, respectively, indicating that in clinical practice, AI-based MRI can be used as an additional tool to help improve the accuracy of preoperative evaluation in individuals diagnosed with endometrial cancer.

The results of this meta-analysis suggest that AI-based MRI demonstrates high diagnostic performance (pooled AUC = 0.90) in predicting cervical stromal invasion, a finding of significant clinical significance. Accurate assessment of cervical stromal invasion is directly related to the choice of surgical approach (whether to undergo radical hysterectomy), yet traditional MRI interpretation is challenging in this regard, relying on the subjective identification of subtle interruptions in the cervical fibrostromal hypointense band and subject to interobserver variability. The study by Gao et al. did not systematically evaluate cervical stromal invasion, while this study is the first to quantitatively synthesize it. Although the sample size is limited, it provides preliminary evidence for the application of AI in this specific field (35). Of particular note is the recent study by Lecointre et al. (2025) (26), which directly compared the diagnostic performance of an AI model with that of human radiologists. The study found that the AI system achieved comparable sensitivity to experienced radiologists in detecting cervical stromal invasion, while exhibiting superior specificity. This suggests that AI may serve as an effective adjunctive tool to reduce overdiagnosis of cervical invasion and avoid unnecessary extensive surgery. However, most current studies have limited sample sizes and are still in the exploratory stage, so we must also view these results with caution. Although the results of this study are encouraging, if AI-based MRI is to be truly integrated into the clinical workflow to assist in the diagnosis of cervical stromal invasion, prospective, large-sample studies are still needed to further verify its superiority over standard imaging interpretation and clarify its ultimate value in improving patient outcomes.

AI has many advantages in the recognition of endometrial cancer MRI images, which has led to its widespread use in clinical practice (36). First, AI can be trained on large amount of MRI data, thereby improving the accuracy and stability of endometrial cancer recognition. By using deep learning and machine learning algorithms to process these data, AI models can learn richer and more complex feature representations, thereby more accurately identifying potential lesions during the diagnosis process (37, 38). This data-driven approach not only improves diagnostic accuracy, but also enables the model to maintain stability and reliability when faced with different types of MRI data (39). Chen et al. (25) conducted a study using 530 MRI images of patients with endometrial cancer and developed a deep learning model. The model was developed to predict the depth of muscle invasion. The results showed that the accuracy of the deep learning model (84.4%) exceeded that of general radiologists (80.0%).

Secondly, AI can automatically extract features and patterns from magnetic resonance images of endometrial cancer, thereby identifying subtle differences that are difficult for doctors to detect. The application of deep learning and machine learning algorithms enables AI models to continuously learn and evolve, thereby continuously improving the accuracy and efficiency of their diagnosis (40). The model can analyze a large amount of case and imaging data to discover patterns and regularities hidden in the data, and apply this information to new imaging data to support doctors in making more accurate diagnoses and assessments (41). In addition, AI offers high efficiency and speed when processing large sets of medical imaging data. It can complete complex analysis tasks related to endometrial cancer in a relatively short time, greatly improving the efficiency and accuracy of diagnosis. Finally, AI can realize automated processes in magnetic resonance imaging recognition, reduce the errors and subjectivity of manual operations, and improve the consistency and repeatability of diagnosis (42). This automation not only improves the accuracy of diagnosis, but also saves doctors valuable time and energy, allowing them to focus more on clinical decision-making and treatment planning. Therefore, the use of AI in MRI interpretation provides important support for improving the efficiency and accuracy of endometrial cancer diagnosis and plays an increasingly important role in clinical practice. However, despite its technical advantages, AI may also have issues such as privacy protection of medical data and compliance with medical regulations. Effectively integrating AI into MRI-based diagnosis of endometrial cancer—while addressing related challenges—may provide clinicians with a valuable tool to improve early detection and treatment. The ultimate goal of this study is to promote the clinical application of AI-assisted decision-making. Although the results show that AI-MRI has good diagnostic performance, its true clinical value lies in whether it can be translated into improved patient management. For example, by more accurately identifying early-stage or low-risk patients, it can help clinicians avoid unnecessary extended surgery or lymph node dissection, thereby reducing surgical complications and improving patients’ quality of life. Future research should focus on comparative studies of “AI vs. doctors” and focus on evaluating the actual impact of AI-assisted decision-making on clinical diagnosis and treatment pathways and patients’ ultimate prognosis. Beyond simply pursuing high diagnostic accuracy, future research must give equal weight to the ‘translational readiness’ of AI models. This includes evaluating model interpretability (e.g., using saliency maps to highlight decision-relevant image regions), computational efficiency, and seamless integration into existing clinical workflows (PACS/RIS). Developing models that are not only accurate but also transparent, efficient, and user-friendly will be essential for gaining clinician trust and facilitating widespread adoption in resource-varying healthcare settings.

This study faces several potential threats to its validity. First, despite employing a comprehensive search strategy, including only English-language literature may lead to language bias, overlooking relevant studies published in other languages. Second, while standardized procedures and arbitration mechanisms exist for study screening and data extraction, researchers’ subjective judgment may still introduce selection and measurement bias. Third, many of the included original studies were retrospective, and some studies had “unclear” areas of risk of bias in the QUADAS-2 quality assessment, which could affect the reliability of the pooled results. Furthermore, clinical and methodological heterogeneity in patient population characteristics, AI model architecture, MRI scan parameters, and interpretation of the gold standard in pathology is not only a major source of statistical heterogeneity but may also threaten the generalizability of the study’s conclusions. Finally, publication bias was detected in the analysis of cervical stromal invasion, indicating that studies with negative results may not have been published, potentially leading to overly optimistic estimates of the AI’s diagnostic performance. These factors collectively threaten the validity of this study’s conclusions, requiring caution in interpreting the results.

This study is not without limitations. First, the majority of the analyzed research consists of retrospective studies, which may introduce inherent and uncontrollable biases. Second, most of the study populations are from Asia and Europe. The generalizability of AI-based MRI for preoperative staging of endometrial cancer to other countries or regions requires confirmation through additional research. Thirdly, the number of original studies included in this meta-analysis is limited, especially the cohorts used to analyze cervical stromal invasion are only 5, and the cohorts used to analyze deep myometrial invasion are 7. The relatively small sample size may affect the stability of the results. More studies need to be included in the future to increase statistical power and verify the current findings. Fourthly, Deeks’ funnel plot asymmetry test suggested potential publication bias in the analysis of cervical stromal invasion (P = 0.02), which may have led to overly optimistic estimates of the diagnostic performance of AI because studies with negative or null results may not have been published. Additionally, the AI ​​models included in this study exhibited significant differences in algorithmic architecture, feature extraction methods, and training processes, which is a major contributor to heterogeneity. This highlights the urgency of standardizing and normalizing AI models in future research. Improving model transparency and reproducibility is crucial to ensuring the comparability of research results and ultimately their translation into clinical practice. Due to limitations in the available data, subgroup analyses based on the model type could not be conducted to identify potential contributors to heterogeneity. In addition, the literature search for this study was closed to March 2025 and was unable to include the latest studies that may have been published thereafter. Future updated analyses need to include more recent results to further verify the conclusions of this study. Finally, some studies included in this meta-analysis used external validation datasets acquired using different imaging equipment and scanning protocols, which could influence both the validation outcomes and contribute to heterogeneity. Future research should involve larger, prospective, and multicenter clinical trials to better assess the utility of AI-based MRI for preoperative staging in endometrial cancer.

## 5 Conclusion

This study demonstrates that AI-based MRI, as an additional tool, can improve the accuracy of preoperative staging of individuals with endometrial cancer to a certain extent. However, due to certain limitations of this study—including small sample sizes, regional bias, retrospective design, and potential publication bias—there is a need for future large-scale, prospective, multicenter clinical trials to further assess the diagnostic utility of AI-based MRI in preoperative staging of endometrial cancer.

## Data Availability

The original contributions presented in the study are included in the article/**Supplementary Material**. Further inquiries can be directed to the corresponding author.
